# Comparison of bioimpedance and anthropometric estimates of body fat in patients with mild autonomous cortisol secretion and nonfunctional adrenal incidentalomas

**DOI:** 10.20945/2359-4292-2026-0040

**Published:** 2026-04-01

**Authors:** Ensar Aydemir, Yasemin Unsal, Coşkun Ates, Semiha Yasar Turk, Soner Cander, Özen Oz Gul, Canan Ersoy, Erdinç Erturk

**Affiliations:** 1 Division of Endocrinology and Metabolism, Edirne Sultan Murat I State Hospital, Edirne, Turkey; 2 Division of Endocrinology and Metabolism, Yıldirim Beyazit University, Yenimahalle Training and Research Hospital, Ankara, Turkey; 3 Division of Endocrinology and Metabolism, Çorlu State Hospital, Tekirdağ, Turkey; 4 Department of Internal Medicine, Muş State Hospital, Muş, Turkey; 5 Division of Endocrinology and Metabolism, Bursa Uludağ University Faculty of Medicine, Bursa, Turkey

**Keywords:** Adrenal incidentaloma, mild autonomous cortisol secretion, body composition, bioelectrical impedance analysis, nonfunctional adrenal incidentaloma

## Abstract

**Objective:**

This study aimed to compare body composition measurements between patients with nonfunctional adrenal incidentalomas (NFAI) and mild autonomous cortisol secretion (MACS) using bioelectrical impedance analysis (BIA) and anthropometric methods.

**Subjects and methods:**

This cross-sectional study included patients diagnosed with MACS or NFAI. Body composition was assessed using BIA, anthropometric measurements, and the Durnin and Womersley (DW) method. Correlation and Bland-Altman analyses were performed to assess the relationship and agreement between the DW method and BIA.

**Results:**

Fifty-seven patients (32 with MACS and 25 with NFAI) were included; those with MACS were older (*p* = 0.004). Post-dexamethasone suppression test cortisol levels (*p* < 0.001) and the incidence of bilateral tumors (*p* = 0.017) were higher in MACS patients. No significant differences in body composition parameters were observed between the MACS and NFAI groups. A strong correlation was observed between BIA- and DW-derived fat mass in MACS patients (*r* = 0.890, *p* < 0.001). Bland-Altman analysis revealed a slight mean bias for body fat mass of -0.4 kg (limits of agreement: -9.14-8.34 kg) and for body fat percentage of -0.83% (limits of agreement: -11.32-9.66%) between methods.

**Conclusion:**

A robust correlation and acceptable agreement was demonstrated between the DW method and BIA for estimating body fat. The DW equation may provide a practical and low-cost alternative for assessing body composition in MACS and NFAI cohorts. Limitations include the lack of a healthy control group and the inability to validate BIA and anthropometric estimates against gold-standard imaging techniques, potentially introducing accuracy bias.

## INTRODUCTION

Adrenal incidentaloma (AI) refers to incidentally detected masses in the adrenal glands during cross-sectional radiological imaging performed for conditions unrelated to the adrenal glands. The prevalence of AI ranges from 1.2% to 8.7%, with rates increasing with age, reaching up to 10% in individuals aged >70 years. ^(1-3)^. Nonfunctional adrenal incidentalomas (NFAIs) account for 40-70% of adrenal adenomas, whereas 30-50% consist of adenomas with mild autonomous cortisol secretion (MACS) ^(1,4,5)^. According to the 2023 ESE/ENAT guidelines, in patients without overt signs and symptoms of Cushing’s syndrome, a morning serum cortisol level exceeding 1.8 µg/dL (>50 nmol/L) after an overnight 1 mg dexamethasone suppression test (DST) is considered indicative of MACS ^(1)^. The increased frequency of cardiometabolic complications in Cushing’s syndrome is well established ^(6)^. Although the association in patients with MACS is not entirely clear, recent studies suggest that chronic cortisol exposure may contribute to an increased risk of diabetes mellitus, hypertension, dyslipidemia, obesity, and mortality ^(7)^.

In individuals with MACS, increased abdominal adiposity is associated with metabolic comorbidities, cardiovascular diseases, and mortality risk ^(4,8)^. The gold-standard methods for assessing abdominal fat are computed tomography and magnetic resonance imaging ^(9)^. Bioelectrical impedance analysis (BIA) is a convenient, cost-effective, and easily applicable method for measuring body composition ^(9)^; however, its reliability has varied across studies ^(9-13)^. Previous studies have evaluated body composition in endocrine disorders using computed tomography, the gold standard ^(4)^. Nevertheless, both computed tomography and dual-energy X-ray absorptiometry are limited by cost and potential radiation exposure ^(9)^. Therefore, our study aimed to investigate how reliably anthropometric measurements reflect visceral adiposity derived from BIA in patients with MACS and NFAI and explore their potential as practical and accessible alternatives in routine endocrinology practice.

## SUBJECTS AND METHODS

### Study population

We conducted a cross-sectional study of body fat distribution ratios obtained through various methods in patients with MACS and NFAI. After ethics committee approval, informed consent was obtained, and patients managed in the endocrinology clinic of a tertiary university hospital between December 1, 2022, and May 31, 2023, were included. Hospital records from January 1, 2019, to May 31, 2023, were screened for patients aged ≥18 years with the diagnosis code “E27.9 Adrenal gland disease, unspecified,” identifying 45 MACS patients. Of these, 32 were contacted by phone and agreed to participate. The NFAI group comprised newly admitted patients. Inclusion criteria were patients with adrenal masses identified on abdominal CT within the previous year. Biochemical and radiological data were retrieved from hospital records. Patients had previously undergone a 1 mg DST, with fasting plasma cortisol levels >1.8 mcg/dL at 8:00 am. denoting MACS. Exclusion criteria were active malignancy, adrenocortical carcinoma, myelolipoma, alcoholism, use of medications affecting dexamethasone metabolism, and history of unilateral or bilateral adrenalectomy. Pheochromocytoma and primary hyperaldosteronism were excluded biochemically and clinically.

### Body composition measurements

Waist, hip, and mid-arm circumferences, as well as skinfold thicknesses at the triceps, subscapular, abdominal, and suprailiac sites, were measured for all participants. Skinfold thicknesses were assessed with a manual caliper; other measurements used a measuring tape. Body fat distribution and lean body mass (LBM) were calculated using the Durnin and Womersley (DW) equation (Online calculator: Durnin and Womersley Calculator). Body composition was also assessed via BIA using the TANITA MC-780 Black. BIA measurements were performed between 8:00 and 10:00 am after at least 8 hours of fasting. Participants stood barefoot, ensuring proper placement of hands and feet on the device sensors. Body mass index (BMI), lean mass, fat mass, fat mass percentage, and muscle mass were automatically calculated.

### Ethical considerations

This study was approved by the local ethics committee (approval no.: 2022-17/31).

### Statistical analysis

Data were expressed as mean ± standard deviation, median with interquartile range (IQR), or frequency and percentage for categorical variables. Pearson’s chi-square test was used to compare categorical variables. The Shapiro-Wilk test assessed normality of continuous variables. Patients were grouped as MACS or NFAI. Continuous variables were analyzed by independent samples t-test or the Mann-Whitney U test, as appropriate. The correlations between overnight 1 mg DST values and body composition measurements were evaluated using Pearson’s correlation analysis. Correlations between body composition measures obtained by the DW method and BIA were also assessed. Agreement between the DW method and BIA measurements was additionally evaluated using Bland-Altman analysis. Statistical analyses were conducted using the IBM SPSS v. 29 (Armonk, USA) software and *p* < 0.05 for statistical significance. Correlation results were visualized using R v. 4.3.2 (2023-10-31 ucrt).

## RESULTS

### Participants and comorbidities

Fifty-seven patients (mean age 58.5 ± 9 years; 45 females, 78.9%) with adrenal adenomas were included. Of these, 32 (56%) were classified as MACS and 25 (44%) as NFAI; NFAI patients were significantly younger than MACS patients (54.6 ± 6.7 vs. 61.5 ± 9.5 years; *p* = 0.004). Hypertension was more frequent in the MACS group (24/32; 75%), and no significant differences were noted in diabetes mellitus or dyslipidemia rates between groups. Cardiovascular disease was present in 5 patients (15.6%) with MACS, while no NFAI patients had cardiovascular disease, although this difference was not statistically significant (*p* = 0.061) (**Table 1**).

**Table 1:** Demographic data and comorbid diseases

Variables	Total (n = 57)	MACS (n = 32)	NFA (n = 25)	*p*
Age (year)	58.5 ± 9	61.5 ± 9.5	54.6 ± 6.7	**0.003**
Sex				
Female, n (%)	45 (78.9)	24 (75)	21 (84)	0.52
Male, n (%)	12 (21.1)	8 (25)	4 (16)	
Height (cm)	162.6 ± 8.2	161.1 ± 8.4	164.6 ± 7.8	0.116
Weight (kg)	81.4 ± 15.9	81.4 ± 16.2	81.5 ± 15.8	0.973
BMI (kg/m^2)^	30.7 ± 5.2	31.4 ± 5.7	29.9 ± 4.4	0.315
BMI classification				
Normal, n (%)	7 (12.3)	4 (12.5)	3 (12)	0.982
Overweight, n (%)	22 (38.6)	12 (37.5)	10 (40)	
Obesity, n (%)	28 (49.1)	16 (50)	12 (48)	
Current smoker, n (%)	20 (35.1)	10 (31.3)	10 (40)	0.35
Alcohol, n (%)	1 (1.8)	1 (3.1)	0	
Comorbidities				
Hypertension, n (%)	31 (54.4)	24 (75)	7 (28)	**0.001**
Diabetes mellitus, n (%)	14 (14)	9 (28.1)	5 (20)	0.691
Dyslipidemia, n (%)	10 (17.5)	7 (21.9)	3 (12)	0.487
Cardiovascular disease, n (%)	5 (8.8)	5 (15.6)	0	0.061
Hypothyroidism, n (%)	14 (24.6)	7 (21.9)	7 (6.1)	0.824

MACS: mild autonomous cortisol secretion; NFA: nonfunctional adenoma; BMI: body mass index.

### Biochemical and radiological parameters

The overnight 1 mg DST results were significantly higher in the MACS group compared to the NFAI group (2.8 [IQR 2.3-3.6] vs. 1.1 [IQR 0.7-1.4], *p* < 0.001), whereas adrenocorticotropic hormone (ACTH) and dehydroepiandrosterone sulfate (DHEAS) levels were lower in the MACS group. Other biochemical parameters were similar between groups (**Table 2**).

**Table 2:** Biochemical parameters of the participants

Variables	Total (n = 57)	MACS (n = 32)	NFA (n = 25)	*p*
1-mg DST (µg/dL)	2.1 (1.2-3)	2.8 (2.3-3.6)	1.1 (0.7-1.4)	**<0.001**
24-hour urine free cortisol (mg/d) (n = 22)	176 (45.4-407)	176 (45.4-407)	-	
ACTH (pg/mL)	13 (6.1-17.8)	10 (6-14)	16 (11.5-20)	**0.007**
DHEA-S (µg/dL)	68.2 (50.3-120.8)	52.6 (35.2-105.3)	85.7 (68.5-138.6)	**0.035**
Fasting glucose (mg/dL)	96 (88.5-114)	94.5 (85.5-113)	99 (89.5-120.5)	0.338
HbA1c (%)	5.7 (5.4-6.1)	5.8 (5.6-6.2)	5.7 (5.3-6.1)	0.329
AST (U/L)	17 (15-22)	18 (15-23)	17 (15-20.5)	0.714
ALT (U/L)	18.4 ± 7.1	18.6 ± 7.6	18.2 ± 6.5	0.856
Creatinine (mg/dL)	0.73 (0.68-0.89)	0.75 (0.7-1)	0.71 (0.64-0.84)	0.06
Sodium (mmol/L)	139 (137-140.5)	139 (137-140)	140 (138-141)	0.329
Potassium (mmol/L)	4.4 ± 0.4	4.4 ± 0.4	4.5 ± 0.5	0.705
Total cholesterol (mg/dL)	213.7 ± 42.2	208.4 ± 44.6	221.6 ± 38	0.273
HDL (mg/dL)	54.7 ± 11.2	52.5 ± 11.8	58 ± 9.5	0.081
LDL (mg/dL)	131.6 ± 37.4	129.5 ± 39.4	134.9 ± 34.7	0.618
Triglycerides (mg/dL)	117.5 (88.5-173)	115 (95-170)	126 (77.5 - 201.5)	0.926
TSH (mU/L)	1.3 (0.7-1.9)	1.24 (0.63-1.61)	1.39 (0.78-2.11)	0.364
Free T4 (ng/dL)	1.01 (0.89-1.09)	1.02 ± 0.14	1.02 ± 0.16	0.883

MACS: mild autonomous cortisol secretion; NFA: nonfunctional adenoma; DST: dexamethasone suppression test; ACTH: adrenocorticotropic hormone; DHEA-S: dehydroepiandrosterone sulfate; HbA1c: hemoglobin A1c; AST: aspartate aminotransferase; ALT: alanine aminotransferase; HDL: high-density lipoprotein; LDL: low-density lipoprotein; TSH: thyroid-stimulating hormone; Free T4: free thyroxine.

Bilateral tumors were detected in 15 (46.9%) MACS patients, significantly more often than in the NFAI group, with only 3 (12%) patients having bilateral tumors (*p* = 0.017). The largest tumor size was similar between groups: 27.5 mm (IQR 20.5-32.7) in MACS and 22 mm (IQR 13-30.5) in NFAI patients (*p* = 0.053) (**Table 3**).

**Table 3:** Radiological characteristics of adrenal adenomas

Variables	Total (n = 57)	MACS (n = 32)	NFA (n = 25)	*p*
Bilateral, n (%)	18 (31.6)	15 (46.9)	3 (12)	**0.017**
Unilateral, n (%)	39 (68.4)	17 (53.1)	22 (88)	
Left, n (%)	26 (45.6)	12 (37.5)	14 (56)	
Right, n (%)	13 (22.8)	5 (15.6)	8 (32)	
Tumor size				
Largest (mm)	26 (19-32)	27.5 (20.5-32.7)	22 (13-30.5)	0.053
Left (mm)	22.4 ± 8.6	23.3 ± 7.8	21 ± 10	0.39
Right (mm)	25 (19-34)	24 (19.2-35)	27 (17-34)	0.967
Tumor density				
Non-contrast (HU) (n = 38)	-5 (-10 ± 5.5)	-5 (-13 ± 1)	-4 (-8 ± 14)	0.123
Contrast-enhanced (n = 5)				
Early phase (HU)	65.2 ± 30.5	38.5 ± 17.7	83 ± 22.9	0.106
Late phase (HU)	25 ± 21.9	23 ± 35	26 ± 18	0.894

MACS: mild autonomous cortisol secretion; NFA: nonfunctional adenoma; HU: Hounsfield unit.

### Body composition measurements

Waist circumference, hip circumference, mid-arm circumference, waist-to-hip ratio, and waist-to-height ratio did not differ significantly between groups. Similarly, there were no significant differences in body fat distribution or LBM values obtained using either the DW equation or the BIA method (**Table 4**).

**Table 4:** Body composition measurements of participants

Variables	Total (n = 57)		MACS (n = 32)	NFA (n = 25)	p
Anthropometric indicators					
Waist circumference (cm)	102.7 ± 12.9		104.9 ± 12.3	99.9 ± 13.5	0.163
Hip circumference (cm)	109.9 ± 10.7		109.7 ± 11.1	110 ± 10.4	0.917
Mid-arm circumference (cm)	30 (28-32)		30.5 (28-32.2)	30 (28.5-32)	0.946
Waist-to-hip ratio (cm/cm)	0.93 ± 0.09		0.96 ± 0.1	0.91 ± 0.08	0.414
Waist-to-height ratio (cm/cm)	0.63 ± 0.08		0.65 ± 0.08	0.61 ± 0.07	0.883
Skinfold thickness					
Biceps (mm)	10.3 ± 3.9		10.6 ± 3.8	10 ± 4.1	0.624
Triceps (mm)	15.4 ± 5.2		16.1 ± 5.4	14.6 ± 4.9	0.317
Subscapular (mm)	21.6 ± 6.4		20.5 ± 5.9	22.9 ± 6.8	0.17
Suprailiac (mm)	16.4 ± 6.2		16 ± 5.8	16.9 ± 6.6	0.601
Body composition					
Durnin and Womersley’s equations					
Body fat (%)	35.7 (32.7-37.5)		35.6 (32.4-37.9)	35.6 (32.6-37.5)	0.939
Body fat mass (kg)	28 ± 8		27.9 ± 8.7	28.1 ± 7.2	0.95
Lean body mass (kg)	53.3 ± 10.4		53.2 ± 10	53.4 ± 10.9	0.935
Bioelectrical impedance analysis					
Body fat (%)	33.3 ± 7.7		34.1 ± 8.8	32.2 ± 5.9	0.35
Body fat mass (kg)	27.6 ± 9.7		28.2 ± 10.4	26.7 ± 8.9	0.566
Lean body mass (kg)	51.9 (47.5-58.5)		50.6 (44.9-56.5)	52.1 (49.6-59.1)	0.563

MACS: mild autonomous cortisol secretion; NFA: nonfunctional adenoma.

### Comparison of body composition measurements

**Figure 1:**
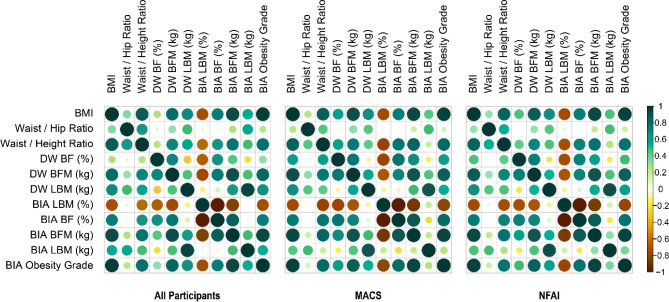
Correlations of body composition measurements. There was a positive and significant correlation in all patients and between BMI and DW BFM (kg) (*r* = 0.861, *p* < 0.001), BIA BFM (kg) (*r* = 0.933, *p* < 0.001), and BIA body fat percentage (BF%) (*r* = 0.748, *p* < 0.001). Positive correlations were also observed between BIA BFM (kg) and DW BFM (kg) in all patients, as well as in the MACS and NFAI groups (*r* = 0.891, *r* = 0.890, *r* = 0.902, *p* < 0.001, respectively). BMI: Body mass index; DW: Durnin-Womersley equation; BF: body fat; BFM: body fat mass; LBM: lean body mass; BIA: bioelectrical impedance analysis; MACS: mild autonomous cortisol secretion; NFAI: nonfunctional adrenal incidentaloma.

Correlations for selected body composition parameters across all methods are shown in **Figure 1**. In the MACS group, there were very strong correlations between body fat distribution (kg) measured by the DW method and BIA (Spearman’s *r* = 0.890, *p* < 0.001).

**Figure 2:**
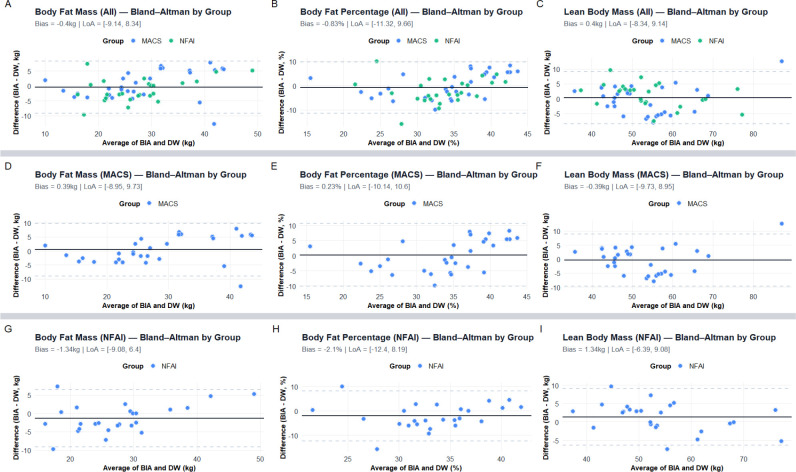
Bland-Altman plots of body composition parameters. Comparisons of bioelectrical impedance analysis (BIA) and the Durnin-Womersley (DW) equation for estimating body composition parameters are depicted. Each plot displays the difference between the two methods (BIA - DW) plotted against their mean, with the solid line indicating the mean bias and dashed lines showing the 95% limits of agreement (LoA). Panels **A-C** show data for all patients; panels **D-F** for those with mild autonomous cortisol secretion (MACS); and panels **G-I** for patients with nonfunctional adrenal incidentaloma (NFAI). Minimal mean bias was observed between BIA and DW across parameters, although the relatively wide LoA suggested moderate inter-individual variability. In the NFAI group, BIA slightly overestimated body fat compared to DW. BIA: bioelectrical impedance analysis; DW: Durnin-Womersley equation; BFM: body fat mass; LBM: lean body mass; MACS: mild autonomous cortisol secretion; NFAI: nonfunctional adrenal incidentaloma; LoA: limits of agreement.

In the overall cohort, Bland-Altman analysis showed minimal mean bias between BIA and the DW equation for body fat mass (-0.4 kg) and LBM (+0.4 kg), with limits of agreement of approximately ±9 kg. Similar results were observed in the MACS group, indicating agreement at the group level. By contrast, the NFAI group showed a slight systematic bias (-1.34 kg for body fat mass and -2.1% for body fat), suggesting that BIA may overestimate body fat relative to the DW method (**Figure 2**). Despite acceptable agreement at the group level, substantial individual-level variability (within ±9-12%) indicates that BIA and DW may not be particularly useful for individual patient monitoring in routine clinical practice.

### Comparison of biochemical parameters with body composition measurements

In the MACS group, the overnight 1 mg DST was significantly correlated with body fat mass percentage by the DW method (*r* = -0.43, *p* = 0.019), whereas no such correlation was observed in the NFAI group. DHEAS levels showed positive correlations exclusively in the NFAI group: with lean body mass (kg) obtained via BIA (*r* = 0.79, *p* = 0.004), body fat mass (kg) obtained via the DW method (*r* = 0.67, *p* = 0.023), and LBM (kg) measured using the DW method (*r* = 0.65, *p* = 0.029).

## DISCUSSION

No significant differences were identified between the MACS and NFAI groups regarding anthropometric measurements, skinfold thicknesses, or body composition measurements obtained using the DW equation and BIA. Some studies have reported an inverse relationship between post-DST cortisol levels and LBM in women, as measured by BIA, while this relationship was not observed in men. Additionally, LBM was similar between NFAI and MACS patients ^(10)^. In one study comparing BIA with dual-energy X-ray absorptiometry, the former underestimated total fat mass compared to the latter in the same individuals ^(11)^. Although research indicates a high correlation between BIA and other methods of body composition analysis, BIA may overestimate LBM and underestimate fat mass, particularly in obese individuals ^(9)^. Considering that approximately half of our patient group consisted of obese individuals, this may explain some of the observed differences. BIA measurements can also be influenced by factors such as age, sex, ethnicity, and medication use ^(9)^. Previous studies have demonstrated a weak correlation between visceral fat mass measurements obtained by BIA and those obtained with computed tomography or magnetic resonance imaging ^(12,13)^.

Some studies have found higher waist-hip ratios in NFAI patients; however, in our measurements, there was no significant difference between groups ^(14,15)^. Garrapa and cols. ^(16)^ reported a positive correlation between waist circumference, waist-hip ratio, and abdominal fat mass. In the present study, a robust correlation was identified between body fat mass measurements obtained using BIA and the DW method, whereas such a correlation was not observed for LBM. This finding underscores the potential utility of skinfold thickness and anthropometric measurements as cost-effective, simple, and practical alternatives for monitoring body fat mass, particularly in settings where access to BIA devices is limited. Consistent with the literature, our study observed that patients in the MACS group were significantly older and predominantly female ^(5,14-17)^. This may be attributed to the fact that MACS patients are often postmenopausal women diagnosed during this period. Additionally, the positive linear relationship between age and overnight DST results may contribute to this observation ^(18)^.

This study demonstrated that there are no significant differences in body composition between NFAI and MACS patients when assessed using anthropometric measurements, the DW equation incorporating various skinfold thicknesses, or the BIA method. When these measurements were compared both across all patients and within the NFAI and MACS groups, significant correlations between methods were observed. Beyond correlational findings, Bland-Altman plots were utilized to evaluate the agreement between BIA and DW methods. The analysis confirmed a substantial degree of inter-method concordance, particularly evident in the MACS cohort. Conversely, a slight systematic bias was observed in the NFAI cohort, which indicated that BIA consistently provided higher estimates of body fat compared to the DW method. Nevertheless, the degree of individual variability suggested that despite showing overall consistent at the group level, these methods may not be sufficiently interchangeable for individual patient monitoring. These findings emphasize that correlation alone is insufficient to establish clinical interchangeability and underscore the necessity of further validation studies utilizing gold-standard imaging modalities (i.e., computed tomography and dual-energy X-ray absorptiometry) ^(11-13)^.

In accordance with the observations reported by Podbregar and cols. ^(17)^, we found no significant disparities in BMI measurements between groups. The study conducted by Olsen and cols. ^(18)^ reported a negative association between BMI values below 30 kg/m^2^ and DST results, whereas no such relationship was identified for BMI values above 30 kg/m^2^. In our study, approximately half of the participants had BMI values above 30 kg/m^2^.

In patients with overt Cushing’s syndrome, there is an increased risk of comorbid conditions such as hypertension, diabetes mellitus, and dyslipidemia. However, the association of these comorbidities with MACS remains uncertain ^(7)^. Some researchers have identified a higher prevalence of comorbid conditions in MACS patients compared to NFAI patients. Furthermore, improvements in these comorbidities, including those observed after adrenalectomy, have been reported in MACS patients ^(5-7,14,19)^. Similar to our findings, several studies did not report consistent or significant differences in the prevalence of complications such as diabetes mellitus and dyslipidemia ^(4,20)^. These differences may be attributed to the saturation of the 11β-HSD2 enzyme in the kidney by cortisol, leading to activation of mineralocorticoid receptors by elevated cortisol levels, subsequently increasing blood pressure. Other potential mechanisms include enhanced beta-adrenergic receptor sensitivity to catecholamines, elevated endothelin-1 levels, or inter-individual variation in glucocorticoid receptor polymorphisms ^(6,21)^. Additionally, the presence of certain comorbidities at similar frequencies in the NFAI group, although not at diagnostic levels, may suggest the possibility of mild cortisol secretion, as supported by prior studies ^(19)^.

As anticipated, in subjects exhibiting autonomous cortisol secretion independent of the hypothalamic-pituitary-adrenal axis, ACTH and DHEAS levels were suppressed and post-DST cortisol levels elevated ^(10,17,20,22)^. This can be explained by the suppression of ACTH resulting from autonomous cortisol release, which, in turn, leads to reduced DHEAS secretion ^(23)^.

In our study, although no significant difference in adenoma size was observed between groups, bilateral adenomas were more frequent in MACS patients, consistent with previous reports. Some studies have shown that adrenal adenoma size is significantly larger in MACS patients and is positively correlated with cortisol levels ^(10,17,20,24)^. Bleier and cols. ^(24)^ reported that an adenoma size smaller than approximately 14 mm had high enough sensitivity to exclude the possibility of MACS. Conversely, Araujo-Castro and cols. ^(25)^ indicated that a size of ≥25 mm had sensitivity and specificity rates up to 70% in favor of MACS. Additionally, bilateral adenomas were associated with higher cortisol levels and lower DHEAS levels ^(3,26,27)^. Nevertheless, some reports have indicated that adenoma size and laterality (unilateral or bilateral) do not provide significantly differentiate between MACS and NFAI ^(5,20,25)^. The lack of differences may not be solely attributable to cortisol secretion but could also be influenced by the secretion of other bioactive metabolites ^(25)^. Moreover, although the BclI polymorphism of the *NR3C1* (glucocorticoid receptor) gene does not appear to affect parameters such as adenoma size, cortisol levels, or BMI, the N363S polymorphism has been reported to be associated with larger tumor sizes in some patients ^(21)^. Furthermore, elevated insulin resistance may stimulate the proliferation of adrenocortical cells, potentially contributing to increased adenoma sizes ^(3)^.

Despite our promising findings, this study has several limitations. First, we were unable to perform computed tomography or magnetic resonance imaging, which are the gold-standard methods for body composition analysis, due to software limitations. Second, the accuracy of BIA measurements may have been compromised by confounding variables such as hydration status, ambient temperature, and concomitant medication use. Third, the overrepresentation of obese patients in the cohort may have resulted in an underestimation of fat tissue measurements derived from BIA. Fourth, although approximate age matching was attempted during data collection, several participants with incomplete anthropometric and biochemical data were necessarily excluded from the final analysis to ensure data integrity. Furthermore, because recruitment efforts were primarily concentrated on patients with MACS, the resulting cohort of NFAI patients was smaller and slightly older on average, leading to group heterogeneity that may have introduced interpretative bias. Fifth, the absence of a healthy control group limited the comparative context and may have provided valuable additional interpretative insight into our findings. Lastly, the relatively lower number of NFAI patients than MACS patients is acknowledged, reflecting the study’s primary focus on MACS during the research period. Future studies should include larger, ageand sex-matched cohorts and use gold-standard imaging modalities.

In conclusion, we demonstrated that there were no significant differences in body composition measurements obtained using BIA between NFAI and MACS patients with AIs. Furthermore, we identified a strong correlation between body fat mass measurements obtained via BIA and the DW method. This suggests that simpler, more accessible, and cost-effective methods beyond BIA analysis may be utilized for body fat mass measurements or follow-up.

## Data Availability

The data that support the findings of this study are available within the article. Due to the sensitive nature of the patient data and ethical restrictions from the hospital system, the raw data are not publicly available but are accessible to qualified researchers upon reasonable request, subject to institutional approval and data usage agreements.
